# Ecosystem Engineers at Work: How Beaver Ponds Reshape Avian Abundance and Diversity in a Mountainous Forest Ecosystem in Central Europe

**DOI:** 10.1002/ece3.74016

**Published:** 2026-07-12

**Authors:** Timo Konstantin Bauer, Stefano Palmero, Eckhard Gottschalk, Marco Heurich

**Affiliations:** ^1^ Department of Conservation Biology University of Göttingen Göttingen Germany; ^2^ Department of National Park Monitoring and Animal Management Bavarian Forest National Park Grafenau Germany; ^3^ Chair of Wildlife Ecology and Management, Faculty of Environment and Natural Resources University of Freiburg Freiburg Germany; ^4^ Institute for Forest and Wildlife Management Campus Evenstad, University of Inland Norway Koppang Norway

**Keywords:** avian diversity, bird counting, ecosystem engineer, Eurasian beaver, freshwater ecosystems

## Abstract

By building dams and creating ponds, the Eurasian beaver ( *Castor fiber* ) acts as an ecosystem engineer, generating habitats for species that depend on wetland and freshwater ecosystems, which are increasingly threatened worldwide. Birds in particular may benefit from these habitats as feeding or breeding grounds. To assess the effects of beaver ponds on avifauna communities, we conducted six 20‐min bird counts between April and June 2021 at 15 beaver ponds and 15 control plots along unmodified streams in the Bavarian Forest National Park, where natural freshwater habitats are scarce. In total, 46 bird species were recorded, of which 13 occurred exclusively at beaver ponds and three only at control sites. Using multiple regression analysis, we investigated the effects of beaver ponds, pond size and a set of environmental variables including habitat openness, standing dead wood and the proportion of deciduous and coniferous trees. Bird abundance was 23% higher at beaver ponds than at control sites. In particular, species associated with aquatic habitats for feeding and breeding occurred at significantly greater abundances at beaver ponds. Species richness and abundance increased with pond surface area, and the largest ponds supported the most distinct bird communities as well as the highest richness and abundance. Moreover, bird community composition differed significantly between beaver ponds and control plots. Our findings demonstrate that beaver‐created wetlands substantially alter bird community composition and enhance habitat availability for wetland‐associated species. These results highlight the important role of beavers as ecosystem engineers in increasing avian diversity and promoting habitat heterogeneity in forest ecosystems.

## Graphical abstract

Eurasian beavers significantly enhance bird diversity and abundance by creating wetland habitats, with 23% higher bird numbers and more unique species at beaver ponds compared to unaffected streams. The size of water surfaces was the key factor influencing species richness, particularly benefiting birds that rely on aquatic environments. Overall, beaver activity reshapes bird communities and increases biodiversity in a mountainous forest ecosystem in Central Europe.

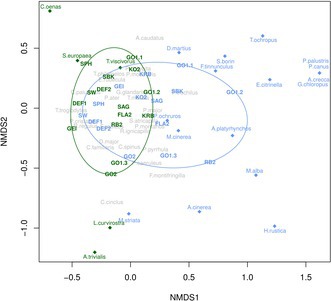

## 1 Introduction

Wetlands and freshwater ecosystems support diverse biological communities and function as important reservoirs of biodiversity (Collen et al. 2014). Over the past century, the global extent of wetlands has been reduced by more than half (Ahmed 2002). Moreover, their quality has deteriorated: in 2010, only 10% of wetlands in the European Union had a favourable conservation status (EEA 2010). The loss of freshwater habitats is subject to five main threats: pollution, modification of water flow, overexploitation, habitat degradation for economic use and exotic species (Dudgeon et al. 2006). Due to the ecological relevance of these areas, these threats directly contribute to biodiversity loss and exacerbate the decline of freshwater species worldwide (Dudgeon et al. 2006; Collen et al. 2014; Reid et al. 2019). While the active restoration of these ecosystems is challenging (Palmer et al. 2014; Moss 2015), the opportunity emerges for passive natural restoration through species acting as ecosystem engineers (Pollock et al. 2014).

Eurasian beavers ( *C. fiber* ), like their North American relative ( *C. canadensis* ), are considered the archetype of ecosystem engineers because they actively manipulate and shape their habitats (Jones et al. 1994; Wright et al. 2002; Sommer et al. 2019). Beavers impound ponds and pools, creating wetlands. In addition, flooding of the perimeter causes shrubs and trees to die off, which, along with tree felling, accumulates dead wood and creates open space. As such, different light and temperature conditions develop. Sediment retention at beaver dams also removes organic material and large amounts of nitrogen from the hydrologic cycle.

The beaver causes changes in water systems, and the establishment of freshwater ponds creates a diverse landscape characterised by heterogeneous structures (Johnston and Naiman 1990; Kivinen et al. 2020). This shaping of watercourses by beavers, most of which have been straightened by humans across Europe, is considered natural restoration (Nummi and Holopainen 2014). Beaver activity has the potential to restore and establish wetlands over time (Law et al. 2017), directly affecting species richness and abundance at both local and landscape scales. Therefore, beavers are considered a keystone species (Wright et al. 2002; Sommer et al. 2019; Orazi et al. 2022). The shift from flowing to standing water provides optimal conditions for the development and dispersal of aquatic invertebrates (Rolauffs et al. 2001; Bush and Wissinger 2016). Fish species benefit from the niches and feeding grounds that are created and therefore increase in diversity, biomass and abundance at beaver‐altered water bodies (Kemp et al. 2012; Needham et al. 2021). On the other hand, migrating fish species can be hindered from reaching their spawning grounds by beaver damming (Needham et al. 2025). Positive effects on the abundance and diversity of reptiles and amphibians have also been reported in association with beaver ponds (Metts et al. 2001; Dalbeck et al. 2007). Furthermore, small mammals use beaver‐made structures for shelter (Samas and Ulevičius 2015). Beaver‐induced standing waters provide optimal hunting conditions for bat species as a result of increased emerging insects (Nummi and Pöysä 1995; Nummi et al. 2011; Orazi et al. 2022). Finally, the abundance of invertebrates, amphibians, small mammals, and fish provides rich foraging grounds for many avian species.

The mosaic‐like heterogeneity of ponds and vegetation creates a high habitat diversity that is particularly used by waterfowl (Grover and Baldassarre 1995; Nummi and Holopainen 2014). These habitats provide the conditions for safe breeding and raising offspring due to the presence of dense riparian vegetation in the floodplains. Various species of ducks use beaver ponds as breeding grounds (Nummi and Pöysä 1997; Nummi and Holopainen 2014). Dense riparian and scrub vegetation on the floodplains and beaver meadows serves as habitat for songbirds, such as shrub‐nesting birds, for example, warblers, which can find here adequate breeding conditions as well as food supplies (Aznar and Desrochers 2008; Fedyń et al. 2023). Shallow water areas along flood zones and pond habitats are frequented by wading or piscivorous birds (Brown et al. 1996; Needham and Kemp 2026). In addition, marsh and reed dwellers such as water rail and moorhen are characteristic avian species at beaver ponds (Grover and Baldassarre 1995). Migrating birds also find suitable stopover sites at beaver habitats, providing shelter and food (Peterson and Low 1977). The dead wood accumulated at the ponds is particularly attractive to woodpeckers, which can forage for insects under the bark or create nest cavities (Grover and Baldassarre 1995; Sikora and Ryś 2004). Beyond wetlands created by beavers, also adjacent terrestrial habitats host a richer and partly distinct breeding bird assemblage compared to unmodified watercourses (Fedyń et al. 2023), with effects detectable up to 100 m into the surrounding forest (Fedyń et al. 2024). As flooding‐induced habitat modification by beavers has a strong effect on local and landscape heterogeneity (Johnston and Naiman 1990; Kivinen et al. 2020), it is expected that habitat diversity is most pronounced at beaver ponds, having the potential to meet the habitat requirements of a diverse range of avian species (Grover and Baldassarre 1995; Fedyń et al. 2023, 2024). This effect was shown to be more pronounced with increasing water surface area, as more ecological niches are available (Brown et al. 1996; Fedyń et al. 2024).

The beaver was widespread in Europe until it was almost extirpated due to overhunting for its fur and meat. Starting with reintroduction projects on the Danube and Isar rivers in the 1960s, the beaver first settled along lowland streams and then returned to upland regions, such as the Bavarian Forest National Park (BFNP) in southeastern Germany. The number of active beaver territories in the BFNP has been growing steadily since the early 2000s. In mountainous landscapes with dense forests, lakes, and water bodies are naturally scarce, beaver activity can play a critical role in avian biodiversity by providing freshwater habitats and wetlands that would not occur locally at this scale (Fedyń et al. 2023, 2024).

This study investigates the consequences of beaver activity on avifauna in the mountainous forest ecosystems of the BFNP, focusing on single ponds. Based on previous research, we hypothesise that (1) beaver ponds enhance avian species richness, (2) avian species richness and species abundance increase with the size of beaver ponds, (3) beaver ponds promote the abundance of avian species associated with water, wetlands and reeds, and (4) beaver ponds support distinct avian species communities.

## 2 Material and Methods

### 2.1 Study Area

The study site was located in the Bavarian Forest National Park (BFNP), in the core of the upland region of the Bavarian Forest, and consists of a strictly protected area of 249 km^2^ along the Bavarian‐Czech border. The Šumava National Park (690 km^2^), the Šumava Protected Landscape Area (996 km^2^) and the Naturpark Bayerischer Wald (2780 km^2^) form the largest contiguous protected forest area in Central Europe, the Bohemian Forest Ecosystem (Heurich et al. 2015). The BFNP reaches elevations of around 600 m.a.s.l. in the valley regions and includes peaks of up to 1.453 m.a.s.l. (Heurich, Beudert, et al. 2010; Heurich et al. 2015). The annual precipitation ranges between 800 and 1.820 mm, and the mean annual temperatures range from 2.0°C to 5.0°C (Heurich, Beudert, et al. 2010).

The BFNP, established in 1970, hosts natural mountain spruce forests (1150 m.a.s.l.), mixed mountain forests (700–1150 m.a.s.l.) and lowland riparian spruce forests (650–900 m.a.s.l.) mainly consisting of Norway spruce ( *Picea abies* ), European beech ( *Fagus sylvatica* ) and White fir ( *Abies alba* ) (Van Der Knaap et al. 2020). Concerning water bodies, several river systems expand throughout the national park, reaching a total length of over 700 km, and natural lakes are scarce.

### 2.2 Selection of Study Sites

**FIGURE 1:**
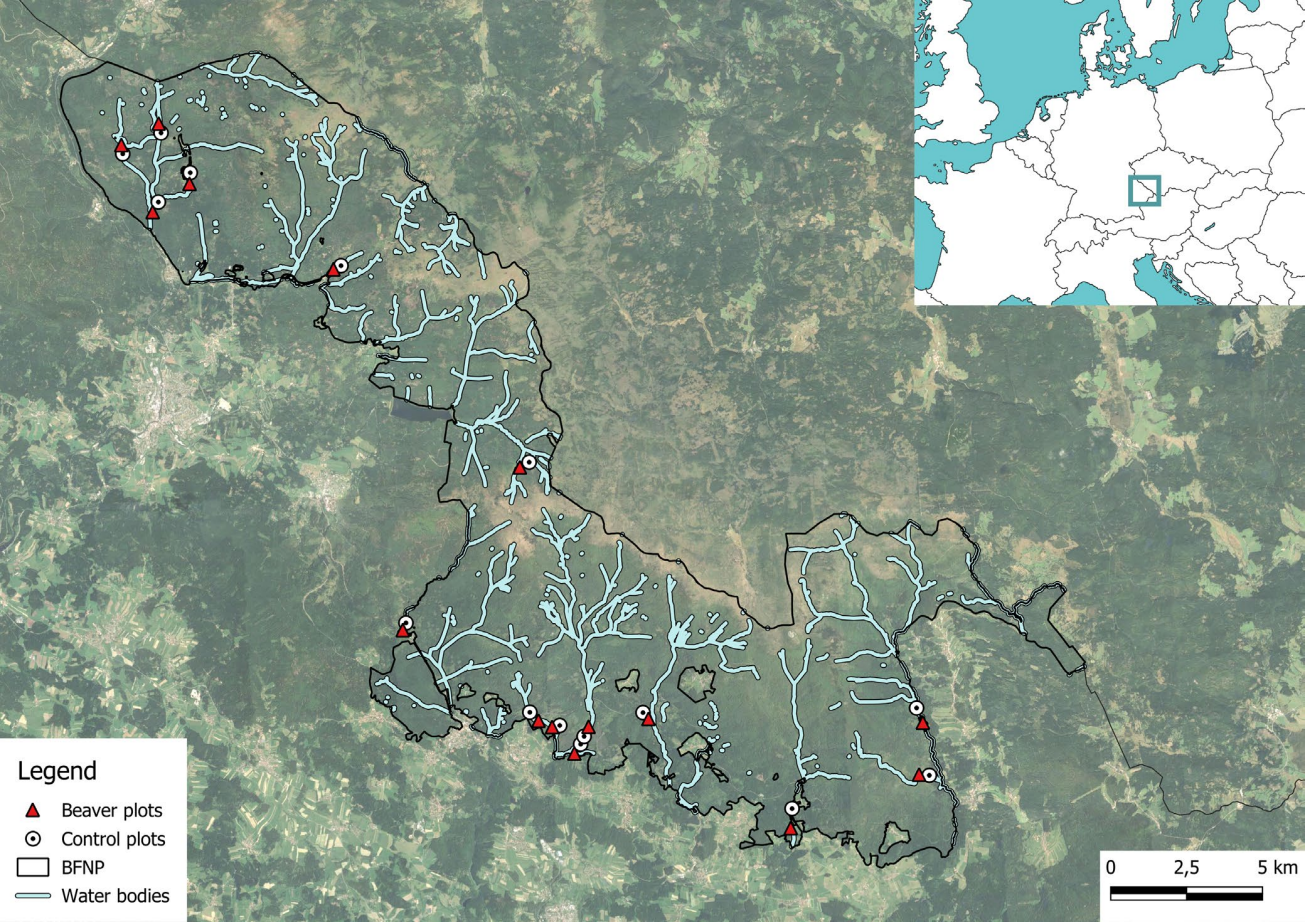
Map of the Bavarian Forest National Park (BFNP) showing the study sites. The beaver plots (red triangles) and the control plots (white dotted circles) are located in pairs near each other. Water bodies are cut within the study area.

**FIGURE 2:**
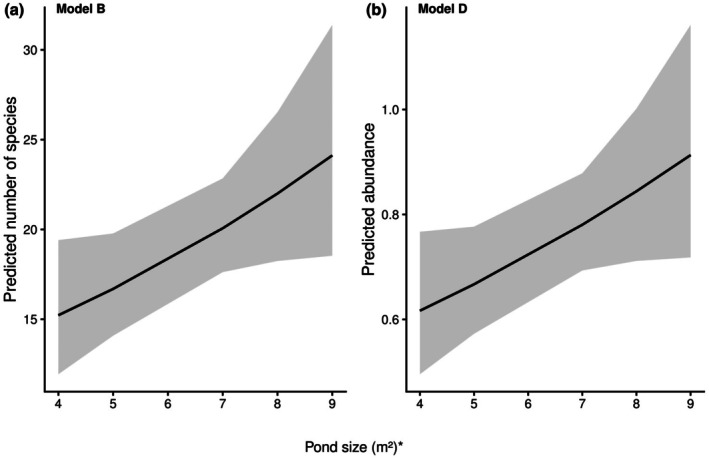
Effect plots (a) predicting species richness (Model B) and (b) abundance (Model D) in relation to pond size. *Pond size values were log‐transformed.

Since 2014, the distribution of the beaver in the national park area has been documented through an annual monitoring programme. In October and November 2020, 22 active beaver territories were recorded at the water bodies in the national park, of which 15 were selected for this study (Figures 1 and 2). Each study site contained a beaver plot, positioned at ponds, and an associated control plot on watercourses unaffected by beavers, for comparison. We aimed to select a gradient of pond sizes, ranging from 80 to 6700 m^2^ (Table S3). The prerequisite for selecting a main beaver plot was the presence of a water body, which had a pond‐like surface structure. Impoundment by raising the water level by means of a dam within a river or stream was not sufficient for this. An increase in the width of the water surface and a significantly reduced flow velocity or stagnant water flow, together with beaver activity, was an essential indication for the selection of the beaver plots. If appropriately distant from each other and with no habitat overlap (minimum 500 m), several beaver plots were also located within a single beaver territory. The same distance was maintained to the standing waters of anthropogenic origin.

In addition to habitat type, environmental parameters such as canopy openness and elevation were matched across beaver and control plots to ensure comparability (Table S3). The centres of the control plots were set, on average, 360 m apart and at least 250 m apart from the centres of the respective beaver plots (Ralph et al. 1995; Bibby et al. 2000).

### 2.3 Bird Monitoring

A plot centre was established on each of the 30 plots as the base for the physical bird survey of the point count. The position was selected according to several criteria. First, having a good overview of the water body, possibly the whole pond, and its immediate surroundings. Since a detection radius of 50 m was chosen for the point count, the centre of the beaver plot had to be close to the pond bank so that the detection range would cover the entire pond. The centre point was marked with a 2 m tall wooden pole. This included both visual and acoustic observations. A pair of binoculars with a 10 × 42 magnification (Leica Trinovid 10 × 42 HD) and identification literature (Svensson 2018) were used for bird identification. Additionally, the minimum number of individuals visible or audible at the same time was noted.

Between 1 April 2021 and 30 June 2021, bird counts were conducted in six survey periods (Table S1). The point‐count method was applied as described by Südbeck et al. (2005) for sampling avifauna. However, instead of their 5‐min sampling time, we used 20 min to cover the majority of the local avifauna at the plots (Blondel et al. 1970). Counts were undertaken either aurally or visually within a 50 m radius from the plot centre. Prerequisites for the counts included good weather conditions, that is, without heavy snowfall, rain or storms. One survey period was defined as two weeks. During these periods, each plot was visited once, and three beaver plots with associated control plots were visited per day. The order of plot visits per survey period remained the same, ensuring a similar time interval between counts at the same plot. Observations were conducted in the early morning hours from sunrise to a maximum of four hours later.

### 2.4 Environmental Variables

The following environmental factors were used as variables for further analysis: standing trees (number of coniferous and deciduous trees, number of standing deadwood), degree of openness (area without canopy cover in ha), and pond size (estimated size in m^2^, only for beaver plots). The forest parameters were derived from LiDAR collected in 2017 for the national park (Yao et al. 2013; Krzystek et al. 2020). Using the geographic information software QGIS (version 3.10.5; QGIS Development Team 2019), the number of standing trees of each tree species, living and dead, and the extent of the canopies were determined over a radius of 50 m around the plot centres (0.785 ha) (Table S2). Since the data capture in 2017, changes have occurred due to bark beetle infestations. We manually updated the dataset using the QGIS software by comparing and adjusting polygons based on the 2021 aerial photo provided by the BFNP administration (Heurich, Ochs, et al. 2010). Using the tracks function of the GPS device GPSMAP 64s from Garmin International Inc. (Olathe, KS, USA), the extent of the pond banks was determined on‐site, after which the pond surface area was calculated (Table S3). In impassable territories, the area was estimated using the 2021 aerial photo and QGIS.

### 2.5 Ecological Guilds

To assess the impact on the abundance of avian species sharing similar main habitats or using the same resources, species were grouped into ecological guilds (Table 1), according to Utschick (2002).

**TABLE 1:** Ecological guilds used in the statistical analysis.

Ecological guild		
Main habitat	Foraging site	Nesting site
Deciduous and mixed forest	Tree	Cavity
Coniferous forest	Ground	Shrub
Hedge, forest edge and succession area	Trunk	Crown
Wetland	Water	Ground and reed
Cultivated landscape		

To allow convergence of statistical models, related guilds with few species or low abundances were grouped (Table S4). Each ecological guild had to be represented by at least two species. The deciduous and mixed deciduous forest birds were grouped with the forest bird generalists. The beaver habitats with wild rivers, streams, gravel pit birds, waterfowl generalists and waterfowl of standing water, banks and siltation zones formed the wetland guild. Since the brambling ( *Fringilla montifringilla* ) was the only species belonging to the group of tundra and taiga birds (as it is a winter visitor species in Germany), it was assigned to its Bavarian refractory habitat group, that is, the cultivated landscape (Utschick 2002). Utschick's scheme was redefined in this study, with a focus on the detailed classification of foraging site guilds. In particular, we simplified them into groups of tree, ground, trunk and water avian species according to their primary foraging habitat. Nesting site guilds remained mostly consistent with Utschick's scheme, except for the ground and reed guilds, which were merged. The white‐throated dipper ( *Cinclus cinclus* ) and the grey wagtail ( *Motacilla cinerea* ) were also included in the latter group, as they are not considered to be building‐breeders in the study area (Table S5).

### 2.6 Statistical Analysis

Based on point counts conducted throughout the survey period, we calculated species richness for each plot as the cumulative number of species recorded. A generalised linear mixed model (GLMM) with a Poisson distribution for count data was fitted in the R package ‘lme4’ (Bates et al. 2015), to evaluate variables determining species richness: plot group, standing deadwood, deciduous trees, coniferous trees and degree of openness (Model A). Due to their proximity to each other and similar confounding effects on beaver and associated control plots, locations were used as a random factor in the model. If required, the environmental factor values were square‐root or log‐transformed to achieve a uniform distribution of the data. In addition, the parameters were *z*‐standardised to ensure comparability between the different units. The influence of the pond size (m^2^) on the species richness of the beaver plots was tested with a generalised linear model (GLM) (Model B). A log transformation was also applied to the values of pond size. A GLM was fitted to elucidate differences in the abundances of all recorded avian species between beaver and control plots (Model C). Subsequently, another GLM was fitted to test the effect of pond size on species abundance (Model D). Abundance values were calculated by summing the maximum number of individuals of each species detected simultaneously per plot across all six survey periods. Species whose total abundance was less than five, summed over all survey periods and plots, were excluded from Model D due to scarce data. Next, a GLMM was used to determine the effect of the plots on the abundance of species assigned to the same ecological guilds, whether they occupy the same main habitats (Model D1), foraging sites (Model D2) or nesting sites (Model D3). In addition to location, avian species were included as a random effect in these models, as individual counts are species‐specific and some species tend to occur in clusters while others are solitary. Hence, this variable is interdependent. Interactions between the variables plot group and ecological guild were included in the models as they affected each other. To visualise the model predictions for species abundances across different ecological guilds at the different plot types, effect plots with Bayesian credible intervals were generated in the R package ‘arm’ (Korner‐Nievergelt and Hüppop 2016; Gelman and Su 2021).

Species that were representative of beaver or control plots due to their abundances could be identified in the R package ‘indicspecies’ (Cáceres and Legendre 2009). The indicator species analysis yields indicator values (IV) for each species, ranging from 0 to 1, and is characterised by the occurrence of each species across the different habitats. Species with values close to 1 are thus highly associated with the respective habitat.

In addition, the distribution of species communities in beaver and control plots was visualised using non‐metric multidimensional scaling (NMDS) in the R package ‘vegan’ (Oksanen et al. 2020), with the Bray–Curtis distance. Significant differences between the species compositions of the two plot communities were determined using the permutational multivariate analysis of variance.

## 3 Results

### 3.1 Species Richness

A total of 46 bird species were recorded during the point counts. In the beaver plots, 43 species were counted, while the control plots had a total species richness of 33. Exclusively at the beaver plots, 13 species were detected, whereas three species were encountered solely at the control plots. At the beaver plots, an average of 18.8 species was recorded, which is 17% more compared to the average of 16.1 at the control plots. Chaffinches ( *F. coelebs* ) and robins ( *Erithacus rubecula* ) were present at all 30 plots. Mallard ( *Anas platyrhynchos* ) and white wagtail ( *M. alba* ) were exclusively recorded at beaver plots. Similarly, the grey wagtail was observed at 11 beaver plots and one control plot. According to the red list of breeding birds of Germany (Ryslavy et al. 2020), the endangered grey‐headed woodpecker ( *Picus canus* ) and the vulnerable Eurasian teal ( *A. crecca* ) were present at the beaver plots. Four near‐threatened species were also detected: spotted flycatcher (*Musciapa striata*), barn swallow ( *Hirundo rustica* ), and common moorhen ( *Gallinula chloropus* ) at the beaver plots and the tree pipit ( *Anthus trivialis* ) at the control plots. The complete list of detected species can be found in Table S6.

From the results of Model A, despite the high number of species found at the beaver plots during the point count, avian species richness was not significantly higher in these plots compared to the control plots. In addition, neither standing trees (coniferous, deciduous, deadwood) nor the degree of openness significantly influenced species diversity (Table S7).

Considering the plots with beaver ponds independently, differences within these plots were evident. Model B results indicated that species richness is positively correlated with the pond surface area. The effect proves to be significant (*p* = 0.04; Table S8). This is illustrated in the effect plot (Figure 2a), which shows the predicted values of species richness in relation to pond size.

### 3.2 Abundance

Due to the rarity and limited coverage of certain species (total abundance < 5), only 31 of the 46 species detected were included in the models. Regardless of avian species, maximum individual numbers shifted at the study sites but were nearly 23% higher at the beaver plots, with a mean of 25.7 compared to the control plots, averaging 20.9 individuals. From Model C, it can be inferred that avian species had significantly higher individual occurrences at the beaver ponds compared to the control plots (*p* = 0.02; Table S9).

Beaver ponds varied in their influence on avian species abundance. The results of Model D indicated that pond size affected the abundance of avian species. Species abundance increased significantly with water surface size (*p* < 0.01; Table S10). The trend line in the effect plot showed an increase in species abundance with increasing pond surface area (Figure 2b).

**FIGURE 3:**
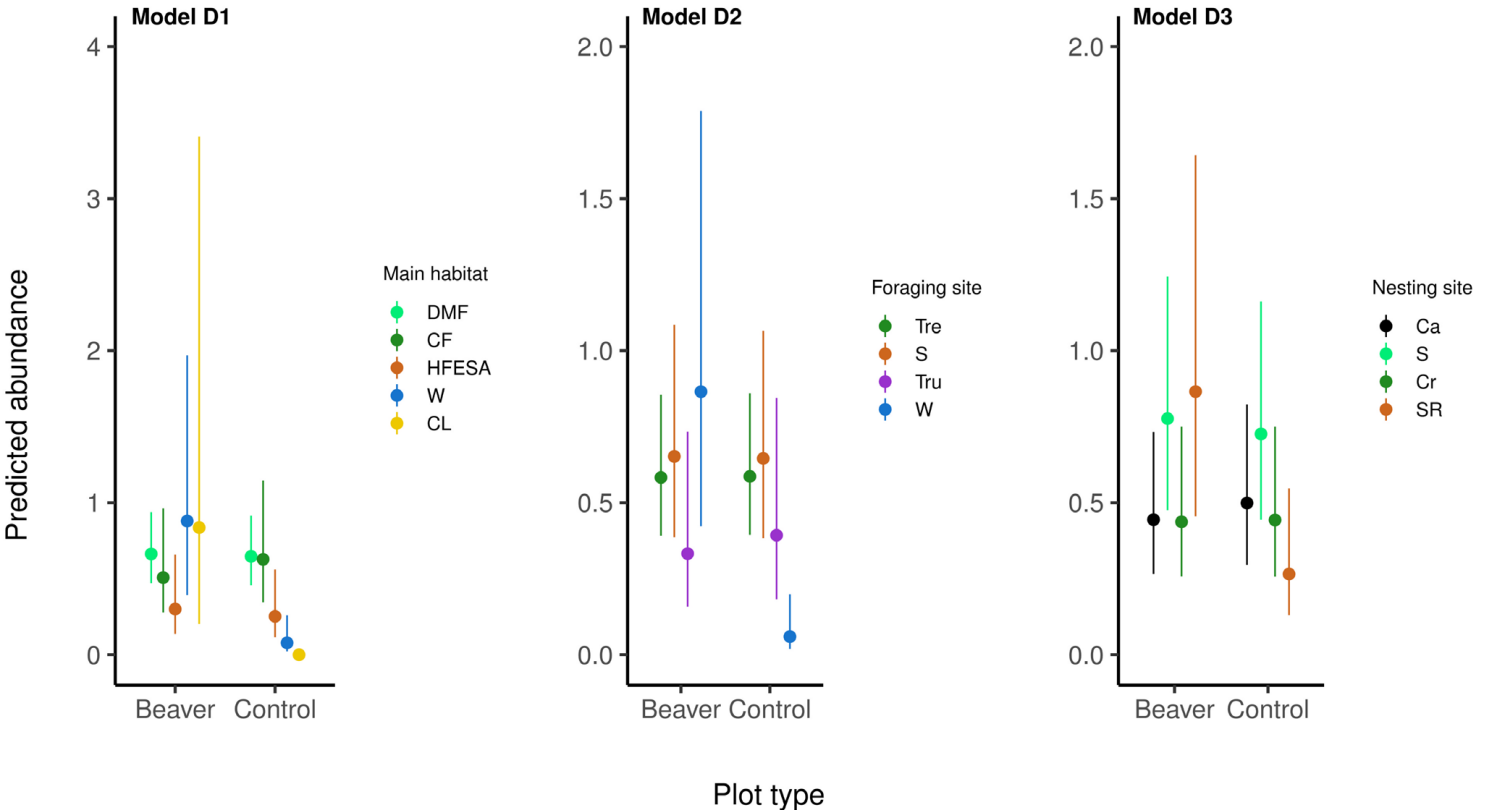
Estimates with 95% credible intervals (CI) of the abundances of species sharing the same main habitats (Model D1), seeking similar locations to forage (Model D2) and similar sites for nesting (Model D3). Concerning ecological guilds, abbreviations were used for spacing reasons. Referring to Table 1, DMF is for deciduous and mixed forest, CF for coniferous forest, HFESA for hedge, forest edge and succession area, W for wetland and CL for cultivated landscape. For the foraging site, Tre refers to tree, G to ground, Tru to trunk, and W to water. Finally, for the nesting sites, Ca refers to cavity, S to shrub, Cr to crown, and GR to ground and reed.

Considering the main habitats, species of the two major guilds had noticeably higher abundances at plots that included a beaver pond (Model D1; Figure 3). Wetland species were significantly more present in the beaver ponds with a predicted mean abundance of 0.89 (*p* < 0.001; Table S11) compared to their occurrences at the control plots (predicted mean = 0.08), indicating a 10‐fold increase. The species of the habitat group cultivated landscape showed a predicted mean abundance of 0.61 (*p* < 0.01; Table S12) at the beaver ponds versus 0.13 at the control plots, which is more than four times higher. Deciduous, mixed, coniferous, and forest edge species abundances did not differ significantly between beaver ponds and control plots in all cases.

Species that prefer to forage on trees, trunks, or on the ground were represented in comparable numbers at beaver ponds and control plots (Model D2; Figure 3). However, the abundance of species foraging in and around water bodies diverged strongly across plot types. With a predicted mean abundance of 0.88, species with this habitat preference had a significant positive association with the beaver ponds (*p* < 0.001; Table S12). The predicted mean abundance of these species in the control plots was way lower at 0.06.

Model D3 revealed that avian species following similar nesting strategies were represented in varying abundances at the different plot types (Figure 3). The abundances of shrub, crown, and cavity‐nesting birds were evenly distributed at the beaver ponds and control plots. Again, one species group showed differences in abundance across plot types. Individuals of species that nest on the ground or in reeds were on average almost certainly present at plots with beaver ponds (predicted mean = 0.91), as confirmed by a significant relationship (*p* < 0.001; Table S13). These species, on the other hand, were sparsely represented in the control plots, leading to a predicted mean abundance of 0.35.

### 3.3 Community Composition

The indicator species analysis showed that three species were significantly associated with the beaver ponds: grey wagtail (*p* = 0.001), mallard (*p* < 0.01) and white wagtail (*p* < 0.01; Table S14). In contrast, no indicator species were found for the control plots. Considering the indicator values, 16 species with an IV > 0.25 could be detected at the beaver ponds. The control plots had five species exceeding this value. Two species tended to be representative for the control plots: mistle thrush (*Turdus viscivorus; p* = 0.072) and Eurasian nuthatch ( *Sitta europaea* ; *p* = 0.069). The remaining 25 species were not associated with beaver ponds or control plots.

**FIGURE 4:**
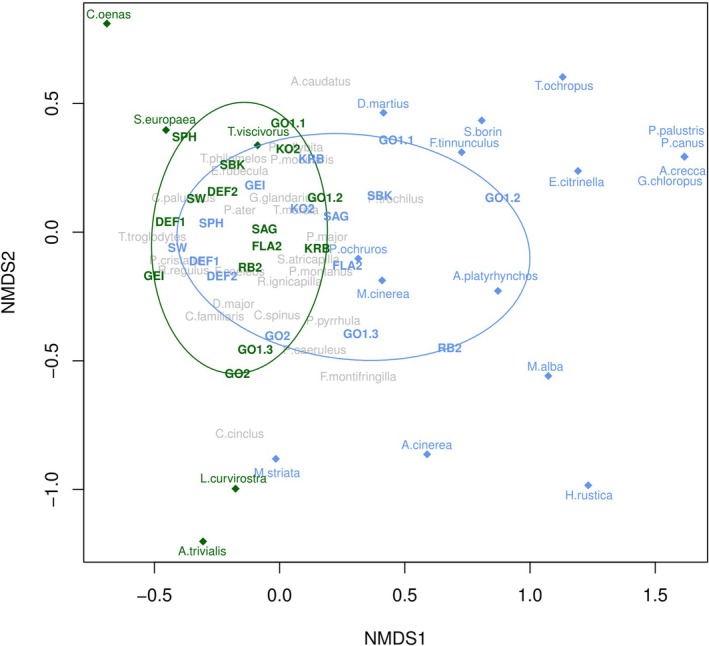
Results of the two‐dimensional NMDS using the Bray‐Curtis distance. Sample plots in capitals. Species names in blue have an IV > 0.25 at the beaver plots. Species names in green have an IV > 0.25 at the control plots. Species names in grey show no association with any plot group. The green circle represents the control plot community, whereas the blue circle shows the beaver plot community. Plot IDs in blue represent the beaver plots, and plot IDs in green represent the respective control plots.

Using NMDS allowed visualisation of differences in species composition and community structure between beaver ponds and control plots (Figure 4). The two‐dimensional distribution of the study plots based on detected species indicated distinct species communities between beaver ponds and control plots. Indeed, the communities of both plot types differed significantly (*p* < 0.01, Table S15). Nevertheless, the species communities overlapped to a certain extent. Notably, beaver pond communities almost entirely covered the control plots' species spectrum.

## 4 Discussion

Our analysis provides evidence that beaver‐created ponds significantly enhanced the avian species communities in a mountainous forest ecosystem in Central Europe. As expected, avian species richness and abundance increase with pond size. Further, species belonging to the wetland guild searching for food in and at water bodies and breeding in reed beds show a higher abundance at the beaver ponds. Finally, the community composition was different at the beaver ponds compared to river sections without beaver activities.

Water surface area proved to be a driving factor of species diversity at the beaver ponds, as already shown in other areas. In Montana, Brown et al. (1996) found a correlation between the area of beaver wetlands and species richness, especially affecting the occurrence of riparian species and ducks and open‐habitat specialists, which were found in beaver ponds larger than 1000 m^2^. Two‐thirds of the ponds in our study had a surface area smaller than 1000 m^2^, which may explain why there was no significant difference in species diversity between beaver and control plots. In recent studies conducted in Poland, across similar mountainous ecosystems, the same correlation was observed (Fedyń et al. 2023, 2024). The authors attribute this to the fact that larger ponds are more heterogeneous due to long‐term beaver activity, characterised by the continual creation and abandonment of dams. This attracts diverse species by providing a wider range of ecological niches (Batáry and Báldi 2004; Stirnemann et al. 2015). However, those studies did not focus on single beaver ponds, but included the entire area around the beaver‐modified water courses. According to Nummi and Holopainen (2014), duck species such as the Eurasian teal and mallard, including offspring sightings of the latter, were found in our study exclusively at water bodies created by beavers. It is considered that particularly the high abundance of insects as a food resource, as well as the shallow water zones used for foraging, provide ducks with an optimal habitat in which they achieve high breeding success (Nummi and Pöysä 1997; Longcore et al. 2006). In addition, other wetland‐related species, such as the grey heron ( *Ardea cinerea* ), common moorhen and green sandpiper ( *Tringa ochropus* ), which used the ponds as a migration stopover or as their foraging habitat, were recorded in such habitats. Species associated with wetlands benefit most from pond size and thus increase the number of more abundant species in larger ponds. Grover and Baldassarre (1995) surveyed the influence of beaver ponds on species richness in 70 wetlands in south‐central New York and found a positive relation between the size of wetlands in active and inactive beaver ponds and avian species richness. Obligately wetland‐dependent species, especially, had a strong association with area size, as observed in more recent studies in Europe (Fedyń et al. 2023, 2024). While wetland size is not equivalent to pond size, beavers generate flood zones and wet meadows when impounding a large pond (Jones et al. 1994; Johnston 2017).

The NMDS results show distinct species communities in beaver ponds and control plots. Noticeable differences in species composition can also be seen within the beaver ponds. The species communities of several beaver ponds resemble those of their respective control plots. In contrast, other beaver ponds show differentiated species communities. Among the beaver ponds, those that deviated most from their respective control plots were those with the largest water surface areas. Surface sizes of ponds at seven beaver ponds exceeded 800 m^2^ (Table S3). Species that were exclusively present or substantially more abundant at the ponds form distinctive species communities that can be clearly distinguished from those of the control plots. Three species were also shown to be indicator species of the beaver ponds, highlighting the impact of beaver‐induced ecosystem modification. While species communities at the control plots are similar and clustered, those at beaver ponds cover a wider range of species. Thus, beaver ponds present a habitat that can support a broad species spectrum (Fedyń et al. 2023). However, apart from pond size, this circumstance may also be linked to other preconditions. Bird densities and species richness at water bodies appear to be greatest when emergent vegetation covers a large portion of the area in addition to open water. In this context, bird populations benefit especially from a 50:50 water‐vegetation cover ratio (Weller and Spatcher 1965). For waterbirds, beavers facilitate entire species communities through habitat diversification, which translates into increased abundance and species richness (Nummi and Holopainen 2014).

While the age of the ponds could not be precisely determined, we observed that several dams were recently constructed. According to time estimates, the territories were on average just under eight years old, with the oldest active territory at 14 years, and several were established in the years before our study took place (Table S3). Due to territorial shifts and the abandonment of individual dams and ponds within the territories, no direct link between the age of the territories and the age and size of the ponds can be inferred. Nevertheless, the environmental impact was much less pronounced at the newly constructed dams and impounded ponds than at older established beaver ponds. However, it has to be considered that several ponds included in this work were potentially too small to significantly alter the ecosystem and consequently provide the habitat diversity needed to support a broad range of species. At their impoundments, pond surfaces were still partially covered with living trees, indicating ongoing flooding effects. Ponds in areas with long‐term beaver activity had more dams with greater lengths. In addition, reeds have already been established on the banks, and wet meadows have developed. This implies that the ponds were in place for a considerable period and that dams were consistently maintained. In the absence of regular maintenance, dams are generally unable to withstand flooding (Pollock et al. 1995). On the other hand, it was reported that the beneficial effects of beaver ponds on bird communities can last even after territories have been abandoned and can even increase due to vegetation dynamics, including advancing succession (Aznar and Desrochers 2008). In northern Minnesota, upon reestablishment after former extirpation, beavers initially colonised areas with the greatest potential for flooding, resulting in large ponds (Johnston and Naiman 1990). After 20 years of progressive population expansion, less suitable patches were selected, resulting in smaller territories and pond sizes because flooding areas were limited. Therefore, beaver dispersal, territory occupancy, and territory characteristics were related to the geomorphological characteristics of the area. These findings can be transferred to our study. Here, the beaver population has also been growing for over 20 years. Thus, the small pond sizes of several ponds selected for this project can be explained by the fact that beavers in the BFNP may now occupy areas less suitable for major flooding, likely due to the steep slopes and rocky river beds in the mountainous landscape.

Species with a primary habitat in water, such as grey wagtails and mallards, were found in significantly greater numbers at the ponds. In addition, white wagtails, which are attributed to the cultural landscape habitat, were significantly more abundant than in the control plots. Since the same species also visit water sites for foraging and build their nesting sites in the direct vicinity of the reeds or on the ground, they occurred in high abundance at the beaver ponds, which were significantly higher than the control plots. This underlines the importance of beaver ponds for species that depend on the ecological conditions of standing water with increased shallow water zones, reedy vegetation along the banks and abundant food sources (Longcore et al. 2006; Nummi and Holopainen 2014; Needham and Kemp 2026). A similar situation applies to species that also occupy wetland‐like habitats but were not included in the model because of their low detection rate. Occurrences of grey heron, green sandpiper, common moorhen, Eurasian teal and common snipe ( *Gallinago gallinago* ; proven by acoustic recordings during the same time period, App. 16), which are threatened by extinction, were recorded only at beaver ponds. In a long‐term monitoring project, investigating habitat changes in beaver territories in Middle Franconia, it was found that over the project period (1999–2018), the number of species and the population density of reed dwellers, water birds and scrubland birds increased (Meßlinger 2009). New colonisations or substantial increases in abundance were found for 50 of the resident avian species. This development was attributed to the water bodies impounded by beaver dams and reed beds formed by flooding over time. Species that had particularly benefited from the beaver activity on the beaver territories in Middle Franconia, such as the red‐backed shrike ( *Lanius collurio* ), could be rediscovered at the ponds in our project via acoustic recordings (App. 16). The species' vocalisations were observed at four beaver ponds. Red‐backed shrikes hunt from perches in semi‐open landscapes, such as at beaver ponds with large floodplains, standing deadwood and reedy vegetation. On the one hand, the increase in water area resulted in habitat diversity, allowing waterfowl to access new and more extensive breeding and feeding areas. The areas also provided feeding habitat for visiting species, as they can find abundant fish and invertebrate resources to feed on. Similarly, reed‐dwelling species were able to find cover in territories with well‐developed reed beds. All these species were found at the beaver ponds in this BFNP, with some being highly abundant. This indicates that water‐ and wetland‐associated as well as reed‐dwelling species occupy and exploit habitats created by beaver activities.

The abundance of species associated with forests or forest edges did not differ between beaver ponds and control plots. A similar outcome was found for species that forage on trees, the ground and tree trunks. Also, species nesting in cavities, shrubs, and tree crowns were equally abundant in the control and beaver ponds. Due to the deadwood accumulation by the beaver, increased use by woodpeckers and other cavity‐dwelling species may be expected. Nevertheless, we did not observe an effect on the abundance of these species. An explanation could be that deadwood is not a scarce resource in the BFNP, which could limit the occurrence of these species at the ponds. In 2002, the BFNP forest inventory reported a deadwood mass of 202 fm/ha (1 Festmeter = 1 cubic metre of solid wood) in the park. This corresponds to 97% of the living wood mass. 70% of the dead biomass occurred as standing deadwood (Heurich and Neufanger 2005). The primary reason for the high concentration of deadwood is the natural disturbance caused by bark beetles in the national park over the last few decades (König et al. 2023). However, it is understood that species that rely on these conditions are provided with an abundance of habitats in the national park and thus may not depend on the areas impacted by the beaver (Beudert et al. 2015). These results should be considered site‐specific to the BFNP, as deadwood frequently presents a limiting resource for avian species elsewhere.

Whether coniferous or deciduous, tree density did not influence the species detected in the plots. The BFNP is dominated by coniferous forests, with spruce as the dominant tree species at all altitudes. Only on the mountain slopes do beech trees occupy a large part of the forest cover and are also sparsely present at the study sites (Cailleret et al. 2014). Species such as the common treecreeper ( *Certhia familiaris* ), coal tit ( *Periparus ater* ) or European crested tit ( *Lophophanes cristatus* ), which are adapted to this predominant habitat type, are common in the region, as confirmed by our results and other studies (Moning and Müller 2008). Due to the uniform presence of coniferous and mixed mountainous forest ecosystems across all study sites and their immediate surroundings, species associated with these habitats were present at the plots in similar numbers.

## 5 Conclusion

Beaver ponds strongly influence avian occurrence and abundance, particularly of water‐associated species, by creating distinct communities that differ from unaffected aquatic habitats. Species diversity, abundance, and community heterogeneity increased with pond size, indicating that pond size, rather than the mere presence of beavers, is the primary determinant of ecological impact.

Large ponds promote long‐term ecosystem changes by maintaining permanent flooding, increasing habitat heterogeneity and creating foraging and breeding opportunities. Damming‐induced flooding drives the development of reed beds, wet meadows, successional vegetation, and dead wood, extending ecological effects into surrounding areas. Accordingly, pond size serves as a robust indicator of the ecological value of beaver activity for avifauna.

Beaver activity particularly benefits species reliant on increasingly rare wetland habitats in European landscapes, including floodplains, natural shorelines, and standing waters. As ecosystem engineers, beavers create and maintain structurally diverse habitats at local and landscape scales, counteracting habitat loss and homogenisation in freshwater and wetland ecosystems.

## Author Contributions

**Timo Konstantin Bauer:** conceptualization (equal), data curation (lead), formal analysis (lead), methodology (equal), writing – original draft (lead), writing – review and editing (equal). **Stefano Palmero:** writing – review and editing (equal). **Eckhard Gottschalk:** conceptualization (equal), investigation (equal), methodology (equal), supervision (equal), writing – review and editing (equal). **Marco Heurich:** conceptualization (equal), investigation (equal), methodology (equal), project administration (lead), resources (lead), supervision (equal), writing – review and editing (equal).

## Funding

This work was supported by Bavarian Forest National Park.

## Conflicts of Interest

The authors declare no conflicts of interest.

## Supporting information


**Table S1:** Overview of survey periods and survey dates of the point count.
**Table S2:** Sampling sheet of the point count.
**Table S3:** Environmental parameters: deadwood (*n*), coniferous trees (*n*), deciduous trees (*n*), area of open space (ha) and estimated pond size (m^2^), elevation, pond size, number of dams, territory foundation year and age.
**Table S4:** Reorganisation of ecological guilds based on Utschick (2002).
**Table S5:** Species in respective ecological guilds. Species in red had a total abundance < 5 and were excluded from the statistical analysis.
**Table S6:** Species list with sum of individual maxima (the highest number of individuals of a species simultaneously observed per plot across all survey periods, added for all respective plots) and abundance per beaver/control plot (mean of the highest number of individuals of a species simultaneously observed per plot across all survey periods) during the point count.
**Table S7:** Results of the generalised linear mixed model (GLMM) on species richness and environmental factors: deadwood, degree of openness, coniferous trees, and deciduous trees (Model A).
**Table S8:** Results of the generalised linear model (GLM) on species richness and pond size (Model B).
**Table S9:** Results of the generalised linear model (GLM) on the effect of the plot types on species abundance (Model C).
**Table S10:** Results of generalised linear model (GLM) on bird abundance and pond size (Model D).
**Table S11:** Results of the generalised linear mixed model (GLMM) on species abundance with predictors of the ecological guilds of the species' main habitat (Model D1). Also, the maximum, mean, and minimum values of the credible intervals of all ecological guilds are shown.
**Table S12:** Results of the generalised linear mixed model (GLMM) on species abundance with predictors of the ecological guilds of the species' primary foraging sites (Model D2). Also, the maximum, mean, and minimum values of the credible intervals of all ecological guilds are shown.
**Table S13:** Results of the generalised linear mixed model (GLMM) on species abundance with predictors of the ecological guilds of the species' primary nesting sites (Model D3). Also, the maximum, mean, and minimum values of the credible intervals of all ecological guilds are shown.
**Table S14:** Indicator species and species with IV values > 0.25 for the respective plot communities. A *p*‐value of 1 is indicated by ‘–’.
**Table S15:** Results of the permutational multivariate analysis of variance via the adonis function of vegan.

## Data Availability

The data that support the findings of this study are openly available in Analysis.zip at https://figshare.com/s/0f30313a66105f33f8b1.
