# Supplementary material for: Ecosystem Engineers at Work: How Beaver Ponds Reshape Avian Abundance and Diversity in a Mountainous Forest Ecosystem in Central Europe
Source: Ecol Evol. 2026 Jul 12;16(7):e74016. doi: 10.1002/ece3.74016 (PMC13357385; doi:10.1002/ece3.74016)
Supplement: Supplementary file 1 — Table S1: Overview of survey periods and survey dates of the point count. Table S2: Sampling sheet of the point count. Table S3: Environmental parameters: deadwood (n), coniferous trees (n), deciduous trees (n), area of open space (ha) and estimated pond size (m2), elevation, pond size, number of dams, territory foundation year and age. Table S4: Reorganisation of ecological guilds based on Utschick (2002). Table S5: Species in respective ecological guilds. Species in red had a total abundance < 5 and were excluded from the statistical analysis. Table S6: Species list with sum of individual maxima (the highest number of individuals of a species simultaneously observed per plot across all survey periods, added for all respective plots) and abundance per beaver/control plot (mean of the highest number of individuals of a species simultaneously observed per plot across all survey periods) during the point count. Table S7: Results of the generalised linear mixed model (GLMM) on species richness and environmental factors: deadwood, degree of openness, coniferous trees, and deciduous trees (Model A). Table S8: Results of the generalised linear model (GLM) on species richness and pond size (Model B). Table S9: Results of the generalised linear model (GLM) on the effect of the plot types on species abundance (Model C). Table S10: Results of generalised linear model (GLM) on bird abundance and pond size (Model D). Table S11: Results of the generalised linear mixed model (GLMM) on species abundance with predictors of the ecological guilds of the species' main habitat (Model D1). Also, the maximum, mean, and minimum values of the credible intervals of all ecological guilds are shown. Table S12: Results of the generalised linear mixed model (GLMM) on species abundance with predictors of the ecological guilds of the species' primary foraging sites (Model D2). Also, the maximum, mean, and minimum values of the credible intervals of all ecological guilds are shown. Table S13: Resu [file ECE3-16-e74016-s001.docx]

**Supplementary material**

**Table S1** Overview of survey periods and survey dates of the point count.

| Survey period | Time frame | Survey days |
| --- | --- | --- |
| 1 | 01/04–15/04 | 02/04, 05/04, 10/04, 11/04, 12/04 |
| 2 | 16/04–30/04 | 22/04, 23/04, 24/04, 25/04, 28/04 |
| 3 | 01/05–15/05 | 04/05, 08/05, 09/05, 10/05, 11/05 |
| 4 | 16/05–30/05 | 21/05, 26/05, 28/05, 29/05, 30/05 |
| 5 | 31/05–14/06 | 02/06, 03/06, 04/06, 08/06, 11/06 |
| 6 | 15/06–30/06 | 15/06, 24/06, 27/06, 28/06, 29/06 |

**Table S2** Sampling sheet of the point count.

| Plot | Survey Period | Date | Time | Weather | °C | Wind | Species | Individuals | Behaviour | Acoustic  detection | Visual  detection | Within  50 m | ≤10 min |
| --- | --- | --- | --- | --- | --- | --- | --- | --- | --- | --- | --- | --- | --- |
|  |  |  |  |  |  |  |  |  |  |  |  |  |  |

**Table S3** Environmental parameters: deadwood (n), coniferous trees (n), deciduous trees (n), area of open space (ha) and estimated pond size (m²), elevation, pond size, number of dams, territory foundation year and age.

| Plot | Deadwood (n) | Conif. trees (n) | Decid. trees (n) | openness (ha) | Estimated pond size (m²) | Coordinates | X (UTM Zone 33) | Y (UTM Zone 33) | Elevation (m) | Dams at the pond | Estimated territory establishment | Age (years) | Number of species |
| --- | --- | --- | --- | --- | --- | --- | --- | --- | --- | --- | --- | --- | --- |
| DEF1 Beaver | 51 | 76 | 37 | 0.27 | 150 | N49° 06.074' E13° 14.118' | 371190 | 5440209 | 702 | 1 | 2011 | 10 | 17 |
| DEF1 Control | 3 | 272 | 25 | 0.10 | – | N49° 05.905' E13° 14.168' | 371243 | 5439895 | 688 | – | – | – | 14 |
| DEF2 Beaver | 2 | 250 | 7 | 0.17 | 80 | N49° 04.507' E13° 13.822' | 370762 | 5437315 | 649 | 1 | 2007 | 14 | 17 |
| DEF2 Control | 1 | 218 | 8 | 0.33 | – | N49° 04.679' E13° 13.991' | 370975 | 5437628 | 646 | – | – | – | 14 |
| FLA2 Beaver | 3 | 62 | 5 | 0.56 | 850 | N48° 56.827' E13° 19.935' | 377890 | 5422918 | 770 | 1 | 2016 | 5 | 23 |
| FLA2 Control | 3 | 139 | 4 | 0.43 | – | N48° 56.950' E13° 20.032' | 378013 | 5423143 | 767 | – | – | – | 14 |
| GEI Beaver | 5 | 188 | 31 | 0.31 | 80 | N49° 04.972' E13° 14.860' | 372046 | 5438147 | 661 | 5 | 2020 | 1 | 20 |
| GEI Control | 2 | 269 | 11 | 0.11 | – | N49° 05.169' E13° 14.897' | 372099 | 5438510 | 689 | – | – | – | 15 |
| GO1.1 Beaver | 10 | 175 | 91 | 0.32 | 1700 | N48° 55.078' E13° 23.453' | 382114 | 5419585 | 751 | 4 | 2010 | 11 | 19 |
| GO126.1 Control | 8 | 208 | 47 | 0.33 | – | N48° 55.236' E13° 23.232' | 381850 | 5419883 | 758 | – | – | – | 17 |
| GO1.2 Beaver | 4 | 44 | 156 | 0.43 | 6700 | N48° 54.953' E13° 23.809' | 382544 | 5419343 | 741 | 2 | 2010 | 11 | 26 |
| GO1.2 Control | 15 | 300 | 45 | 0.24 | – | N48° 54.971' E13° 24.017' | 382798 | 5419371 | 751 | – | – | – | 20 |
| GO1.3 Beaver | 40 | 118 | 30 | 0.37 | 1300 | N48° 54.458' E13° 24.367' | 383206 | 5418412 | 740 | 2 | 2010 | 11 | 22 |
| GO1.3 Control | 2 | 100 | 2 | 0.57 | – | N48° 54.617' E13° 24.551' | 383437 | 5418702 | 736 | – | – | – | 21 |
| GO2 Beaver | 55 | 166 | 4 | 0.44 | 400 | N48° 54.920' E13° 24.791' | 383741 | 5419257 | 741 | 1 | 2010 | 11 | 24 |
| GO2 Control | 1 | 66 | 2 | 0.58 | – | N48° 54.751' E13° 24.656' | 383570 | 5418948 | 738 | – | – | – | 16 |
| KO2 Beaver | 9 | 218 | 22 | 0.32 | 700 | N48° 54.998' E13° 26.418' | 385731 | 5419360 | 733 | 3 | 2013 | 8 | 20 |
| KO2 Control | 4 | 219 | 16 | 0.32 | – | N48° 55.113' E13° 26.280' | 385567 | 5419577 | 720 | – | – | – | 14 |
| KRB Beaver | 29 | 194 | 35 | 0.32 | 500 | N48° 59.600' E13° 23.345' | 382160 | 5427965 | 1076 | 1 | 2018 | 3 | 14 |
| KRB Control | 2 | 131 | 54 | 0.37 | – | N48° 59.681' E13° 23.605' | 382480 | 5428108 | 1034 | – | – | – | 16 |
| RB2 Beaver | 5 | 29 | 8 | 0.70 | 1200 | N48° 54.642' E13° 33.801' | 394734 | 5418524 | 789 | 2 | 2012 | 9 | 13 |
| RB2 Control | 3 | 92 | 3 | 0.60 | – | N48° 54.907' E13° 33.656' | 394566 | 5419018 | 795 | – | – | – | 19 |
| SAG Beaver | 36 | 139 | 30 | 0.34 | 1600 | N48° 52.907' E13° 30.075' | 390120 | 5415397 | 710 | 1 | 2011 | 10 | 21 |
| SAG Control | 1 | 167 | 28 | 0.26 | – | N48° 53.251' E13° 30.142' | 390215 | 5416033 | 740 | – | – | – | 17 |
| SW Beaver | 2 | 143 | 24 | 0.32 | 150 | N49° 05.730' E13° 13.075' | 369907 | 5439601 | 683 | 2 | 2013 | 8 | 14 |
| SW Control | 12 | 93 | 77 | 0.25 | – | N49° 05.571' E13° 13.101' | 369931 | 5439306 | 674 | – | – | – | 14 |
| SPH Beaver | 1 | 115 | 52 | 0.22 | 70 | N49° 03.310' E13° 18.619' | 376552 | 5434964 | 777 | 1 | 2020 | 1 | 12 |
| SPH Control | 4 | 89 | 92 | 0.19 | – | N49° 03.365' E13° 18.830' | 376810 | 5435059 | 812 | – | – | – | 13 |
| SBK Beaver | 9 | 149 | 11 | 0.52 | 450 | N48° 53.722' E13° 33.615' | 394474 | 5416824 | 797 | 1 | 2018 | 3 | 20 |
| SBK Control | 5 | 176 | 89 | 0.19 | – | N48° 53.697' E13° 33.890' | 394809 | 5416771 | 786 | – | – | – | 17 |

**Table S4** Reorganisation of ecological guilds based on Utschick (2002).

| Category | Main habitat | Main habitat | Foraging site | Foraging site | Nesting site | Nesting site |
| --- | --- | --- | --- | --- | --- | --- |
| Ecological guilds | Deciduous and mixed forest | Forest generalists | Tree | Whole wooded area | Cavity | Cavity breeders |
|  |  | Deciduous and mixed forest |  | Crown area | Shrub | Shrub breeders |
|  | Coniferous forest | Coniferous forest |  | No preference | Crown | Crown breeders |
|  | Hedge, forest edge, succession area | Forest edge, succession, hedge, park, orchard | Ground | Ground, herbaceous layer | Ground and reed | Ground breeders |
|  | Wetland | Wild river, stream, gravel pit |  | Herbaceous layer, reed, undergrowth |  | Reed breeders |
|  |  | Waterfowl Generalists | Trunk | Trunk, inner crown |  | Building breeders |
|  |  | Stillwaters, banks, siltation zones | Water | Water |  | |
|  | Cultivated landscape | Cultivated landscape |  | Water/reed/ground |  |  |

**Table S5** Species in respective ecological guilds. Species in red had a total abundance <5 and were excluded from the statistical analysis.

| Main Habitat | Deciduous and mixed forest | Coniferous forest | Hedge, forest edge, succession area | Wetland | Cultivated landscape |
| --- | --- | --- | --- | --- | --- |
|  | Turdus merula | Loxia curvirostra | Anthus trivialis | Motacilla cinerea | Motacilla alba |
|  | Parus caeruleus | Parus cristatus | Carduelis spinus | Ardea cinerea | Fringilla montifringilla |
|  | Fringilla coelebs | Regulus ignicapillus | Phylloscopus trochilus | Anas crecca | Phoenicurus ochruros |
|  | Dendrocopos major | Parus ater | Sylvia borin | Anas platyrhynchos | Hirundo rustica |
|  | Garrulus glandarius | Certhia familiaris | Pyrrhula pyrrhula | Gallinula chloropus | Falco tinnunculus |
|  | Picus canus | Regulus regulus | Emberiza citrinella | Tringa ochropus |  |
|  | Prunella modularis |  | Muscicapa striata | Cinclus cinclus |  |
|  | Columba oenas |  |  |  |  |
|  | Sitta europaea |  |  |  |  |
|  | Parus major |  |  |  |  |
|  | Turdus viscivorus |  |  |  |  |
|  | Sylvia atricapilla |  |  |  |  |
|  | Columba palumbus |  |  |  |  |
|  | Erithacus rubecula |  |  |  |  |
|  | Aegithalos caudatus |  |  |  |  |
|  | Dryocopus martius |  |  |  |  |
|  | Turdus philomelos |  |  |  |  |
|  | Parus palustris |  |  |  |  |
|  | Parus montanus |  |  |  |  |
|  | Troglodytes troglodytes |  |  |  |  |
|  | Phylloscopus collybita |  |  |  |  |
| Foraging site | Tree | Ground | Trunk | Water | Air |
|  | Parus caeruleus | Turdus merula | Dendrocopos major | Motacilla alba |  |
|  | Fringilla coelebs | Anthus trivialis | Columba oenas | Motacilla cinerea | Hirundo rustica |
|  | Garrulus glandarius | Fringilla montifringilla | Sitta europaea | Ardea cinerea |  |
|  | Carduelis spinus | Emberiza citrinella | Dryocopus martius | Anas crecca | No Preference |
|  | Loxia curvirostra | Picus canus | Certhia familiaris | Anas platyrhynchos |  |
|  | Phylloscopus trochilus | Phoenicurus ochruros |  | Gallinula chloropus | Falco tinnunculus |
|  | Sylvia borin | Prunella modularis |  | Tringa ochropus |  |
|  | Pyrrhula pyrrhula | Turdus viscivorus |  | Cinclus cinclus |  |
|  | Muscicapa striata | Columba palumbus |  |  |  |
|  | Parus cristatus | Erithacus rubecula |  |  |  |
|  | Parus major | Turdus philomelos |  |  |  |
|  | Sylvia atricapilla | Troglodytes troglodytes |  |  |  |
|  | Aegithalos caudatus |  |  |  |  |
|  | Regulus ignicapillus |  |  |  |  |
|  | Parus palustris |  |  |  |  |
|  | Parus ater |  |  |  |  |
|  | Parus montanus |  |  |  |  |
|  | Regulus regulus |  |  |  |  |
|  | Phylloscopus collybita |  |  |  |  |
| Nesting site | Cavity | Shrub | Crown | Ground and Reed | Building |
|  | Parus caeruleus | Turdus merula | Anthus trivialis | Motacilla alba | Hirundo rustica |
|  | Dendrocopos major | Sylvia borin | Fringilla montifringilla | Phylloscopus trochilus | Phoenicurus ochruros |
|  | Muscicapa striata | Pyrrhula pyrrhula | Garrulus glandarius | Motacilla cinerea |  |
|  | Picus canus | Emberiza citrinella | Carduelis spinus | Anas crecca |  |
|  | Parus cristatus | Prunella modularis | Loxia curvirostra | Anas platyrhynchos |  |
|  | Columba oenas | Turdus viscivorus | Ardea cinerea | Gallinula chloropus |  |
|  | Sitta europaea | Sylvia atricapilla | Columba palumbus | Cinclus cinclus |  |
|  | Parus major | Erithacus rubecula | Aegithalos caudatus | Phylloscopus collybita |  |
|  | Dryocopus martius | Turdus philomelos | Regulus ignicapillus |  |  |
|  | Parus palustris | Troglodytes troglodytes | Falco tinnunculus |  |  |
|  | Parus ater |  | Tringa ochropus |  |  |
|  | Certhia familiaris |  | Regulus regulus |  |  |
|  | Parus montanus |  | Fringilla coelebs |  |  |

**Table S6** Species list with sum of individual maxima (the highest number of individuals of a species simultaneously observed per plot across all survey periods, added for all respective plots) and abundance per beaver/ control plot (mean of the highest number of individuals of a species simultaneously observed per plot across all survey periods) during the point count.

| Species | | Status | Control plot | | Beaver plot | |
| --- | --- | --- | --- | --- | --- | --- |
|  |  |  | Sum of individual maxima | Abundance | Sum of individual maxima | Abundance |
| Mallard | Anas platyrhynchos | LC | 0 | 0.00 | 26 | 1.70 |
| Common chaffinch | Fringilla coelebs | LC | 25 | 1.70 | 25 | 1.70 |
| Eurasian blackcap | Sylvia atricapilla | LC | 21 | 1.40 | 24 | 1.60 |
| European robin | Erithacus rubecula | LC | 20 | 1.30 | 24 | 1.60 |
| Willow warbler | Phylloscopus trochilus | LC | 13 | 0.90 | 18 | 1.20 |
| Coal tit | Periparus ater | LC | 23 | 1.50 | 17 | 1.10 |
| Common chiffchaff | Phylloscopus collybita | LC | 19 | 1.30 | 17 | 1.10 |
| Common blackbird | Turdus merula | LC | 12 | 0.80 | 17 | 1.10 |
| Eurasian wren | Troglodytes troglodytes | LC | 18 | 1.20 | 15 | 1.00 |
| Great tit | Parus major | LC | 14 | 0.90 | 15 | 1.00 |
| Song thrush | Turdus philomelos | LC | 14 | 0.90 | 15 | 1.00 |
| Grey wagtail | Motacilla cinerea | LC | 1 | 0.10 | 15 | 1.00 |
| Dunnock | Prunella modularis | LC | 15 | 1.00 | 13 | 0.90 |
| Eurasian siskin | Carduelis spinus | LC | 14 | 0.90 | 13 | 0.90 |
| White wagtail | Motacilla alba | LC | 0 | 0.00 | 13 | 0.90 |
| European crested tit | Lophophanes cristatus | LC | 13 | 0.90 | 11 | 0.70 |
| Common firecrest | Regulus ignicapilla | LC | 9 | 0.60 | 11 | 0.70 |
| Common wood pigeon | Columba palumbus | LC | 6 | 0.40 | 9 | 0.60 |
| Eurasian treecreeper | Certhia familiaris | LC | 11 | 0.70 | 8 | 0.50 |
| Goldcrest | Regulus regulus | LC | 10 | 0.70 | 8 | 0.50 |
| Eurasian bullfinch | Pyrrhula pyrrhula | LC | 5 | 0.30 | 8 | 0.50 |
| Great spotted woodpecker | Dendrocopos major | LC | 6 | 0.40 | 7 | 0.50 |
| Eurasian jay | Garrulus glandarius | LC | 6 | 0.40 | 6 | 0.40 |
| Brambling | Fringilla montifringilla | LC | 4 | 0.30 | 6 | 0.40 |
| Willow tit | Poecile montanus | LC | 4 | 0.30 | 5 | 0.30 |
| Black woodpecker | Dryocopus martius | LC | 1 | 0.10 | 5 | 0.30 |
| White-throated dipper | Cinclus cinclus | LC | 3 | 0.20 | 4 | 0.30 |
| Garden warbler | Sylvia borin | LC | 1 | 0.10 | 4 | 0.30 |
| Mistle thrush | Turdus viscivorus | LC | 9 | 0.60 | 3 | 0.20 |
| European blue tit | Cyanistes caeruleus | LC | 2 | 0.10 | 3 | 0.20 |
| Black redstart | Phoenicurus ochruros | LC | 0 | 0.00 | 3 | 0.20 |
| Eurasian nuthatch | Sitta europaea | LC | 8 | 0.50 | 2 | 0.10 |
| Yellowhammer | Emberiza citrinella | LC | 0 | 0.00 | 2 | 0.10 |
| Spotted flycatcher | Musciapa striata | V | 0 | 0.00 | 2 | 0.10 |
| Eurasian teal | Anas crecca | 3 | 0 | 0.00 | 2 | 0.10 |
| Barn swallow | Hirundo rustica | V | 0 | 0.00 | 2 | 0.10 |
| Green sandpiper | Tringa ochropus | LC | 0 | 0.00 | 2 | 0.10 |
| Long-tailed tit | Aegithalus caudatus | LC | 3 | 0.20 | 1 | 0.10 |
| Grey heron | Ardea cinerea | LC | 0 | 0.00 | 1 | 0.10 |
| Grey-headed woodpecker | Picus canus | 2 | 0 | 0.00 | 1 | 0.10 |
| Marsh tit | Poecile palustris | LC | 0 | 0.00 | 1 | 0.10 |
| Common moorhen | Gallinula chloropus | V | 0 | 0.00 | 1 | 0.10 |
| Common kestrel | Falco tinnunculus | LC | 0 | 0.00 | 1 | 0.10 |
| Common crossbill | Loxia curvirostra | LC | 2 | 0.10 | 0 | 0.00 |
| Tree pipit | Anthus trivialis | V | 1 | 0.10 | 0 | 0.00 |
| Stock dove | Columba oenas | LC | 1 | 0.10 | 0 | 0.00 |

**Table S7** Results of the generalised linear mixed model (GLMM) on species richness and environmental factors: deadwood, degree of openness, coniferous trees, and deciduous trees (Model A).

| Predictors | Species Richness | | | |
| --- | --- | --- | --- | --- |
|  | Incidence rate ratios | SE | CI | p |
| (Intercept) | 16.40 | 1.13 | 14.33–18.77 | <0.001 |
| Beaver plot | 1.12 | 0.11 | 0.92–1.37 | 0.26 |
| Deadwood | 1.03 | 0.05 | 0.93–1.13 | 0.62 |
| Degree of openness | 1.04 | 0.08 | 0.89–1.21 | 0.61 |
| Coniferous trees | 0.99 | 0.06 | 0.87–1.13 | 0.92 |
| Deciduous trees | 1.01 | 0.06 | 0.90–1.14 | 0.83 |
| Random effects | | | | |
| σ2 | 0.06 | | | |
| Τ00 plot | 0.00 | | | |
| Nplot | 15 | | | |
| Observations | 30 | | | |
| Marginal R2/conditional R2 | 0.130/NA | | | |

**Table S8** Results of the generalised linear model (GLM) on species richness and pond size (Model B).

| Predictors | Species Richness | | | |
| --- | --- | --- | --- | --- |
|  | Incidence rate ratios | SE | CI | p |
| (Intercept) | 10.53 | 3.14 | 5.83–18.74 | <0.001 |
| Pond size | 1.10 | 0.05 | 1.00–1.20 | 0.04 |
| Observations | 15 | | | |
| R2 nagelkerke | 0.398 | | | |

**Table S9** Results of the generalised linear model (GLM) on the effect of the plot types on species abundance (Model C).

| Predictors | Abundance | | | |
| --- | --- | --- | --- | --- |
|  | Incidence Rate Ratios | SE | CI | p |
| (Intercept) | 0.66 | 0.04 | 0.59–0.74 | <0.001 |
| Beaver plot | 1.20 | 0.09 | 1.03–1.39 | 0.02 |
| Observations | 930 | | | |
| R2 Nagelkerke | 0.009 | | | |

**Table S10** Results of generalised linear model (GLM) on bird abundance and pond size (Model D).

| Predictors | Abundance | | | |
| --- | --- | --- | --- | --- |
|  | Incidence Rate Ratios | SE | CI | p |
| (Intercept) | 0.40 | 0.11 | 0.24 – 0.67 | 0.001 |
| Pond size | 1.11 | 0.04 | 1.03 – 1.20 | <0.01 |
| Observations | 465 | | | |
| R2 Nagelkerke | 0.024 | | | |

**Table S11** Results of the generalised linear mixed model (GLMM) on species abundance with predictors of the ecological guilds of the species’ main habitat (Model D1). Also, the maximum, mean, and minimum values of the credible intervals of all ecological guilds are shown.

| Predictors | Abundance | | | |
| --- | --- | --- | --- | --- |
|  | Incidence Rate Ratios | SE | CI | p |
| (Intercept) | 0.66 | 0.10 | 0.49–0.89 | <0.01 |
| Beaver Plot | 1.02 | 0.10 | 0.85–1.24 | 0.80 |
| Coniferous forest | 1.26 | 0.39 | 0.68–2.31 | 0.46 |
| Hedge, forest edge, succession area | 0.71 | 0.26 | 0.35–1.44 | 0.34 |
| Wetland | 0.12 | 0.07 | 0.04–0.39 | <0.001 |
| Cultivated landscape | 0.19 | 0.12 | 0.05–0.68 | 0.01 |
| Beaver plot * coniferous forest | 0.81 | 0.17 | 0.54–1.22 | 0.31 |
| Beaver plot * hedge, forest edge,  succession area | 1.27 | 0.32 | 0.78–2.07 | 0.34 |
| Beaver plot * Wetland | 10.98 | 5.76 | 3.92–30.72 | <0.001 |
| Beaver plot * cultivated landscape | 4.63 | 2.55 | 1.57–13.65 | <0.01 |
| Random Effects | | | | |
| σ2 | 0.96 | | | |
| T00 species | 0.29 | | | |
| T00 plot | 0.01 | | | |
| ICC | 0.24 | | | |
| Nplot | 15 | | | |
| Nspecies | 31 | | | |
| Observations | 930 | | | |
| Marginal R2/conditional R2 | 0.192/0.387 | | | |

**Table S12** Results of the generalised linear mixed model (GLMM) on species abundance with predictors of the ecological guilds of the species' primary foraging sites (Model D2). Also, the maximum, mean, and minimum values of the credible intervals of all ecological guilds are shown.

| Predictors | Abundance | | | |
| --- | --- | --- | --- | --- |
|  | Incidence Rate Ratios | SE | CI | p |
| (Intercept) | 0.68 | 0.11 | 0.50–0.92 | 0.01 |
| Beaver Plot | 1.03 | 0.11 | 0.84–1.27 | 0.75 |
| Ground | 1.09 | 0.28 | 0.65–1.81 | 0.75 |
| Trunk | 0.59 | 0.21 | 0.29–1.19 | 0.14 |
| Water | 0.09 | 0.05 | 0.03–0.28 | <0.001 |
| Beaver plot * ground | 1.01 | 0.18 | 0.72–1.42 | 0.97 |
| Beaver plot * trunk | 0.82 | 0.25 | 0.45–1.49 | 0.51 |
| Beaver plot * water | 14.03 | 7.31 | 5.05–38.97 | <0.001 |
| Random Effects | | | | |
| σ2 | 0.96 | | | |
| T00 species | 0.27 | | | |
| T00 plot | 0.01 | | | |
| ICC | 0.23 | | | |
| N plot | 15 | | | |
| N species | 31 | | | |
| Observations | 930 | | | |
| Marginal R2/conditional R2 | 0.24/0.41 | | | |

**Table S13** Results of the generalised linear mixed model (GLMM) on species abundance with predictors of the ecological guilds of the species’ primary nesting sites (Model D3). Also, the maximum, mean, and minimum values of the credible intervals of all ecological guilds are shown.

| Predictors | Abundance | | | |
| --- | --- | --- | --- | --- |
|  | Incidence Rate Ratios | SE | CI | p |
| (Intercept) | 0.51 | 0.11 | 0.33–0.78 | <0.01 |
| Beaver plot | 0.89 | 0.14 | 0.65–1.22 | 0.46 |
| Shrub | 1.45 | 0.43 | 0.81–2.60 | 0.22 |
| Crown | 1.21 | 0.39 | 0.64–2.28 | 0.55 |
| Ground and reed | 0.69 | 0.25 | 0.35–1.39 | 0.30 |
| Beaver plot * shrub | 1.20 | 0.25 | 0.80–1.79 | 0.37 |
| Beaver plot * crown | 1.18 | 0.27 | 0.76–1.84 | 0.45 |
| Beaver plot * ground and reed | 2.90 | 0.73 | 1.77–4.75 | <0.001 |
| Random Effects | | | | |
| σ2 | 0.96 | | | |
| T00 species | 0.30 | | | |
| T00 plot | 0.01 | | | |
| ICC | 0.24 | | | |
| N plot | 15 | | | |
| N species | 31 | | | |
| Observations | 930 | | | |
| Marginal R2/conditional R2 | 0.057/0.288 | | | |

**Table S14** Indicator species and species with IV values >0.25 for the respective plot communities. A p-value of 1 is indicated by “–“.

| Study Site | Species | IV value | p |
| --- | --- | --- | --- |
| Beaver Plot | Motacilla cinerea | 0.844 | 0.001 |
|  | Anas platyrhynchos | 0.730 | 0.002 |
|  | Motacilla alba | 0.683 | 0.004 |
|  | Dryocopus martius | 0.527 | 0.20 |
|  | Sylvia bo | 0.492 | 0.15 |
|  | Emberiza citrinella | 0.365 | 0.47 |
|  | Phoenicurus ochruros | 0.365 | 0.47 |
|  | Tringa ochropus | 0.365 | 0.51 |
|  | Ardea cinerea | 0.258 | – |
|  | Musciapa striata | 0.258 | – |
|  | Picus canus | 0.258 | – |
|  | Anas crecca | 0.258 | – |
|  | Hirundo rustica | 0.258 | – |
|  | Poecile palustris | 0.258 | – |
|  | Gallinula chloropus | 0.258 | – |
|  | Falco tinnunculus | 0.258 | – |
| Control Plot | Turdus viscivorus | 0.647 | 0.07 |
|  | Sitta europaea | 0.624 | 0.07 |
|  | Anthus trivialis | 0.258 | – |
|  | Loxia curvirostra | 0.258 | – |
|  | Columba oenas | 0.258 | – |

**Table S15** Results of the permutational multivariate analysis of variance via the adonis function of vegan.

|  | Df | Sums of Sqs. | Mean Sqs. | F.Model | R2 | Pr (>F) | Sig. |
| --- | --- | --- | --- | --- | --- | --- | --- |
| Plot type | 1 | 0.2723 | 0.27230 | 27.018 | 0.088 | 0.009 | ** |
| Residuals | 28 | 28.220 | 0.10078 | – | 0.912 | – | – |
| Total | 29 | 30.943 | – | – | 1.000 | – |  |
